# Comparative Genomic Study of Lactobacillus jensenii and the Newly Defined Lactobacillus mulieris Species Identifies Species-Specific Functionality

**DOI:** 10.1128/mSphere.00560-20

**Published:** 2020-08-12

**Authors:** Catherine Putonti, Jason W. Shapiro, Adriana Ene, Oleksandra Tsibere, Alan J. Wolfe

**Affiliations:** a Department of Biology, Loyola University Chicago, Chicago, Illinois, USA; b Bioinformatics Program, Loyola University Chicago, Chicago, Illinois, USA; c Department of Microbiology and Immunology, Stritch School of Medicine, Loyola University Chicago, Maywood, Illinois, USA; University of Michigan—Ann Arbor

**Keywords:** *Lactobacillus*, *Lactobacillus jensenii*, *Lactobacillus mulieris*, urinary microbiome, urogenital microbiome

## Abstract

Lactobacilli are dominant members of the “healthy” female urogenital microbiota. One of these species, Lactobacillus jensenii, is routinely identified in the urinary microbiota of women both with and without urinary tract symptoms. In March 2020, the new bacterial species Lactobacillus mulieris was introduced, and phylogenetic and average nucleotide identity analysis identified eight L. jensenii strains that should be classified as members of the L. mulieris species. This prompted our phylogenomic study of all publicly available L. jensenii and *L. mulieris* genome sequences. While there is little variation in the 16S rRNA gene sequences, the core genome shows a clear distinction between genomes of the two species. We find eight additional strains of the species *L. mulieris* among these genomes. Furthermore, one strain, currently classified as *L. mulieris* UMB7784, is distinct from both L. jensenii and *L. mulieris* strains. As part of our comparative genomic study, we also investigated the genetic content that distinguishes these two species. Unique to the L. jensenii genomes are several genes related to catabolism of disaccharides. In contrast, *L. mulieris* genomes encode several cell surface and secreted proteins that are not found within the L. jensenii genomes. These L. jensenii-specific and *L. mulieris*-specific loci provide insight into phenotypic differences of these two species.

**IMPORTANCE** *Lactobacillus* species play a key role in the health of the urinary tract. For instance, Lactobacillus crispatus and L. jensenii have been found to inhibit uropathogenic Escherichia coli growth. While L. crispatus is typically found only within the microbiota of women without lower urinary tract symptoms (LUTS), L. jensenii has been found in the microbiota of women both with and without LUTS. With the recent introduction of the new species *Lactobacillus mulieris*, several strains of L. jensenii were reclassified as *L. mulieris* based upon gene marker and average nucleotide identity. We took a phylogenomic and comparative genomic approach to ascertain the genetic determinants of these two species. Looking at a larger data set, we identified additional *L. mulieris* strains, including one distinct from other members of the species—*L. mulieris* UMB7784. Furthermore, we identified unique loci in each species that may have clinical implications.

## Abstract (precis)

*Lactobacillus* species play a key role in the health of the urinary tract. For instance, Lactobacillus crispatus and L. jensenii have been found to inhibit uropathogenic Escherichia coli growth. While L. crispatus is typically found only within the microbiota of women without lower urinary tract symptoms (LUTS), L. jensenii has been found in the microbiota of women both with and without LUTS. With the recent introduction of the new species *Lactobacillus mulieris*, several strains of L. jensenii were reclassified as *L. mulieris* based upon gene marker and average nucleotide identity. We took a phylogenomic and comparative genomic approach to ascertain the genetic determinants of these two species. Looking at a larger data set, we identified additional *L. mulieris* strains, including one distinct from other members of the species—*L. mulieris* UMB7784. Furthermore, we identified unique loci in each species that may have clinical implications.

## OBSERVATION

The *Lactobacillus* species L. crispatus, L. gasseri, L. iners, and L. jensenii are predominant members of the “healthy” female urogenital microbiota (1, 2). L. jensenii has been shown to be a protective species, reducing growth of uropathogenic Escherichia coli and sexually transmitted infections (3–6). Recently, Rocha et al. (7) presented a new *Lactobacillus* species, L. mulieris. The type strain for this new species is *L. mulieris* c10Ua161M, isolated from a urine sample. Rocha et al. (7) found that the *L. mulieris* c10Ua161M genome, with eight L. jensenii strains, had an average nucleotide identity (ANI) with the L. jensenii type strain genome below the 95% species threshold (8). Coinciding with the Rocha et al. publication (7), we deposited and published two new *Lactobacillus* urinary isolates (9, 10); while matrix-assisted laser desorption ionization–time of flight mass spectrometry (MALDI-TOF MS) classified these strains as L. jensenii, ANI analysis suggested they were members of the new *L. mulieris* species. This prompted us to investigate 43 publicly available L. jensenii and *L. mulieris* genomes (see Table S1 in the supplemental material).

10.1128/mSphere.00560-20.3 TABLE S1 Metadata for the 43 genomes examined in this study. The isolation site and host health status listed are based upon GenBank annotation. Bladder indicates that the sample was from a catheterized urine specimen, while urinary tract indicates that the urine collection method was not specified. Download Table S1, DOCX file, 0.02 MB. Copyright © 2020 Putonti et al. 2020 Putonti et al. This content is distributed under the terms of the Creative Commons Attribution 4.0 International license.

**FIG 1:**
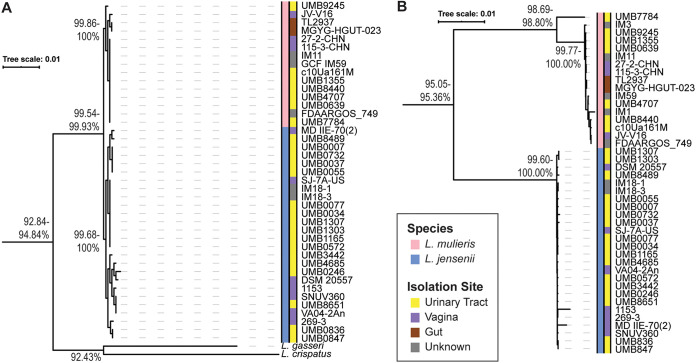
Comparison of L. jensenii and L. mulieris genomes. (A) 16S rRNA gene sequence comparison. L. gasseri ATCC 33323 = JCM 1131 (GenBank accession no. NC_008530) and L. crispatus ST1 (NC_014106) are included as an outgroup. The range of pairwise identity between groups is shown on the branches. (B) Phylogenetic tree of single-copy-number genes in the core genome. The range of pairwise identity between groups is shown on the branches. In the legend or key, the colors used for the species designation determined in this study and the isolation site are indicated for all genomes in both trees.

First, we examined the L. jensenii and *L. mulieris* 16S rRNA gene sequences, which were ≥99.54% identical (Fig. 1A). The two species can be distinguished by just two nucleotides in the 16S rRNA gene sequence (see Fig. S1 in the supplemental material). The *L. mulieris* clade included the type strain, *L. mulieris* c10Ua161M, as well as strains identified by Rocha et al. (7) as *L. mulieris* based upon *pheS* and *rpoA* gene sequence trees. Our two recent sequences UMB7784 and UMB9245, as well as six other strains, form a clade with the *L. mulieris* type strain, suggesting that they too represent this new species.

10.1128/mSphere.00560-20.2 FIG S1 Alignment of 16S rRNA gene sequences for L. jensenii and *L. mulieris strains*. In total, 13 positions in the alignment were discordant between the sequences. Strains having the same nucleotide at these positions are shown in the same color (red or black). “Lj” indicates the nucleotide at the position in *L. jensenii* strains. “Lm” indicates the nucleotide at the position in *L. mulieris* strains. Nucleotides at these positions are listed for the aligned 16S rRNA sequences of *L. gasseri* ATCC 33323 = JCM 1131 (NC_008530) (“Lg”) and *L. crispatus* ST1 (NC_014106) (“Lc”). Download FIG S1, PDF file, 0.1 MB. Copyright © 2020 Putonti et al. 2020 Putonti et al. This content is distributed under the terms of the Creative Commons Attribution 4.0 International license.

Next, the pangenome and set of single-copy genes in the core genome of the 43 L. jensenii and *L. mulieris* genomes were identified using the tool anvi’o (11) (see Text S1 in the supplemental material). Phylogenomic analysis is based upon the concatenated protein sequences of the 453 single-copy core genes found, and sequence identities are reported for these concatenated sequences (Fig. 1B). This tree showed clear distinction between genomes of the two species. Pairwise amino acid sequence comparisons between L. jensenii and *L. mulieris* core genomes ranged from 95.05 to 95.36% sequence identity. In contrast, the core genome sequence of the 26 genomes that form a clade with the L. jensenii type strain are 99.60% to 100% identical. On the basis of this core genome analysis, we propose that 17 strains should be classified as members of the species *L. mulieris* (Table S1).

10.1128/mSphere.00560-20.1 TEXT S1 Supplemental methods. Download Text S1, DOCX file, 0.03 MB. Copyright © 2020 Putonti et al. 2020 Putonti et al. This content is distributed under the terms of the Creative Commons Attribution 4.0 International license.

The variation within the *L. mulieris* clade of the core genome tree is primarily due to one isolate—*L. mulieris* UMB7784 (10). The core genome of strain UMB7784 is less similar to other *L. mulieris* strains than they are to each other. Using JSpeciesWS (12), we compared the two species’ type strains to *L. mulieris* UMB7784. *L. mulieris* UMB7784 had an ANI value of 87.81% to L. jensenii DSM 20557 and 96.29% to *L. mulieris* c10Ua161M. Excluding UMB7784, the *L. mulieris* strains have an average ANI value of 99.66% (Table S2). While UMB7784 is best grouped with *L. mulieris*, additional sequencing of *L. mulieris* strains may reveal a third group to which it belongs.

10.1128/mSphere.00560-20.4 TABLE S2 ANIb values for the 17 *L. mulieris* genomes examined in this study. Highlighted is UMB7784, the most divergent strain of the species. An asterisk indicates identity. Values were calculated by JSpeciesWS (Richter and Rosselló-Móra [12]). Download Table S2, DOCX file, 0.02 MB. Copyright © 2020 Putonti et al. 2020 Putonti et al. This content is distributed under the terms of the Creative Commons Attribution 4.0 International license.

**FIG 2:**
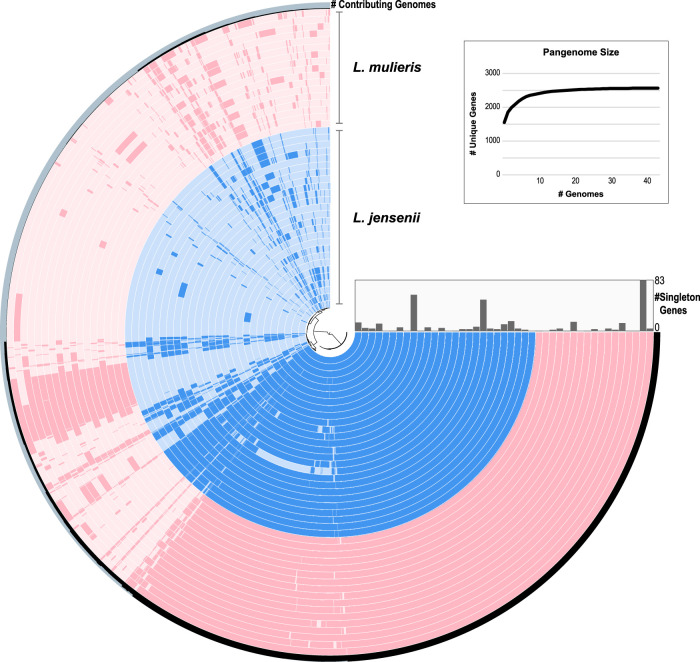
Pangenome analysis of 26 L. jensenii (blue) and 17 *L. mulieris* (pink) genomes. Each ring corresponds to a single genome, and each radial extension in the ring corresponds to a particular gene. If the gene is present in the genome, the color is darker than if it is absent from the genome. The outer ring includes the percentage of the genomes including the gene. The three largest number of singleton genes from the center out are L. jensenii UMB0077, L. jensenii UMB8651, and *L. mulieris* UMB7784. The inset shows the increase in the pangenome size as new genomes are examined.

The accessory genome for these strains includes 1,738 gene clusters (Fig. 2), which is larger than the core genome. *L. mulieris* UMB7784 contains the most genes (*n *= 83) that are unique to a single genome (singleton genes). Prior genome analyses of lactobacilli found that the genus has an open pangenome (13, 14). Species that colonize diverse habitats and/or coexist with other microbes in large communities, such as many *Lactobacillus* species, typically have open pangenomes (15). While the majority of comparative genome studies of lactobacilli have focused on those species most relevant to the dairy industry, a recent analysis of L. paragasseri and L. gasseri considered isolates from the human gut microbiota (16). In addition to inhabiting the gut microbiota, *L. paragasseri* and L. gasseri are common inhabitants of the female urogenital tract, and Zhou et al. (16) found that they also have an open pangenome. Our pangenome analysis of the L. jensenii and *L. mulieris* strains finds that they too have an open pangenome.

Last, we investigated the genetic content that distinguishes these two species. We examined each species individually and identified genes conserved among all genomes of that species that were not present in genomes of the other species. This analysis found 32 genes for L. jensenii (Table S3) and 62 genes for *L. mulieris* (Table S4). Some L. jensenii genes support the findings of Rocha et al. (7). For instance, L. jensenii can use ribose and trehalose, while *L. mulieris* cannot; only the L. jensenii genomes encode a ribose transporter and trehalose operon repressor (Table S3). Rocha et al. (7) also noted that only L. jensenii can use arbutin. Our genome analysis identified a L. jensenii locus for disaccharide catabolism. Further investigation would be needed to ascertain whether this is arbutin. We also found new signs of functional specificity, including a conserved respiratory chain pathway present only in L. jensenii (Table S3) and multiple cell surface and secreted proteins in *L. mulieris* (Table S4). While none of these *L. mulieris*-specific genes are found in a L. jensenii strain, some of these genes do have homologs within L. psittaci, L. crispatus, and *L. paragasseri* (based on blastp to the nr database).

10.1128/mSphere.00560-20.5 TABLE S3 Genes conserved and unique to L. jensenii strains. Genome positions listed are for the coding region locations within the genome of L. jensenii SNUV360 (GCF_001936235). The product accession refers to the protein product annotated for this genome. Download Table S3, DOCX file, 0.01 MB. Copyright © 2020 Putonti et al. 2020 Putonti et al. This content is distributed under the terms of the Creative Commons Attribution 4.0 International license.

10.1128/mSphere.00560-20.6 TABLE S4 Genes conserved and unique to *L. mulieris* strains. Genome positions listed are for the coding region locations within the genome of *L. mulieris* c10Ua161M (GCF_007095465). As this assembly is not a single chromosome sequence, the contig containing the coding region is also listed. The product accession refers to the protein product annotated for this genome. Download Table S4, DOCX file, 0.02 MB. Copyright © 2020 Putonti et al. 2020 Putonti et al. This content is distributed under the terms of the Creative Commons Attribution 4.0 International license.

While other lactobacilli of the urinary tract have been associated with or without lower urinary tract symptoms (LUTS), L. jensenii has been found in communities regardless of urinary symptoms (17). As our 16S rRNA gene sequence analysis suggests, distinguishing between L. jensenii and *L. mulieris* by 16S amplicon sequencing surveys is error-prone. Thus, whole-genome sequencing of the microbiota or directed searches for the genes identified here (Tables S3 and S4) is likely the best way to distinguish the two species in a community. With this knowledge, we can determine whether L. jensenii and/or *L. mulieris* have any association with LUTS. Furthermore, previous studies associating L. jensenii with the benefit of inhibiting the growth of urogenital pathogens must be revisited to assess whether both species have this beneficial role. The distinction of these two species may explain reported phenotypic variation among L. jensenii isolates, including H_2_O_2_ production (18, 19). Twenty-three of the 43 genomes examined here are from our own collection of urinary isolates, and none of our 6 *L. mulieris* strains were isolated from women without LUTS (Table S1).

Recently, the entire *Lactobacillus* genus has been reevaluated given the availability of numerous complete and draft genome sequences (20, 21). Our phylogenetic and phylogenomic analyses support the reclassification of the eight L. jensenii genomes identified by Rocha et al. (7) and eight additional genomes into the new *L. mulieris* species. Furthermore, we found that the *L. mulieris* UMB7784 genome is distinct from other *L. mulieris* strains. Looking at the 83 genes unique to this strain (Table S5), we found homologs in other lactobacilli, including L. salivarius, L. crispatus, L. hominis, L. johnsonii, and *L. psittaci*. UMB7784-specific genes include genes that encode the following metabolism-related proteins or pathways: glucose phosphotransferase system (PTS), arginase, glucocerebrosidase, diaminopropionate ammonia-lyase, and a phospholipase and a phosphate-selective porin. The UMB7784-specific genes also include genes encoding proteins related to survival: dTDP-4-dehydrorhamnose 3,5-epimerase, a bacteriocin, and a protein G-related albumin-binding molecule. *L. mulieris* UMB7784 suggests that the urogenital tract may contain additional lactobacilli subspecies or species. Further investigation of lactobacilli from the urogenital tract will provide critical insight into the genomic diversity of these two species and potential associations with urogenital symptoms and infections.

10.1128/mSphere.00560-20.7 TABLE S5 Genes conserved and unique to *L. mulieris* UMB7784. The best blastp hit as well as the best blastp hit to a *Lactobacillus* genome is listed for each anvi’o gene cluster identified as UMB7784-specific. Download Table S5, XLSX file, 0.01 MB. Copyright © 2020 Putonti et al. 2020 Putonti et al. This content is distributed under the terms of the Creative Commons Attribution 4.0 International license.
