# Supplementary material for: Comparative Genomic Study of Lactobacillus jensenii and the Newly Defined Lactobacillus mulieris Species Identifies Species-Specific Functionality
Source: mSphere. 2020 Aug 12;5(4):e00560-20. doi: 10.1128/mSphere.00560-20 (PMC7426171; doi:10.1128/mSphere.00560-20)
Supplement: TABLE S1 [file mSphere.00560-20-st001.docx]

| **Strain** | **Isolation site [host health status]** | **Species** | **RefSeq Accession #** | **CheckM Completeness** | **CheckM Contamination** |
| --- | --- | --- | --- | --- | --- |
| 1153 | Vagina [unknown] | *L. jensenii* | GCF_000155915 | 100 | 16.1 |
| SJ-7A-US | Vagina [healthy] | *L. jensenii* | GCF_000162335 | 100 | 10 |
| 269-3 | Vaginal cavity [healthy] | *L. jensenii* | GCF_000175035 | 100 | 3.6 |
| MD IIE-70(2) | Vagina [healthy] | *L. jensenii* | GCF_000466805 | 100 | 16.2 |
| IM18-1 | Unknown | *L. jensenii* | GCF_001012665 | 100 | 6.9 |
| IM18-3 | Unknown | *L. jensenii* | GCF_001012685 | 100 | 6.9 |
| DSM 20557 | Vaginal discharge [unknown] | *L. jensenii* | GCF_001436455 | 100 | 6.9 |
| SNUV360 | Vaginal environment [healthy] | *L. jensenii* | GCF_001936235 | 100 | 3.6 |
| UMB0077 | Bladder (female) [OAB] | *L. jensenii* | GCF_002848045 | 100 | 3.6 |
| UMB0007 | Bladder (female) [no LUTS] | *L. jensenii* | GCF_002863405 | 100 | 3.6 |
| UMB0732 | Bladder (female) [no LUTS] | *L. jensenii* | GCF_007785825 | 99.9 | 3.8 |
| UMB0037 | Bladder (female) [no LUTS] | *L. jensenii* | GCF_007785915 | 100 | 3.6 |
| UMB8489 | Bladder (female) [rUTI] | *L. jensenii* | GCF_007785935 | 100 | 3.6 |
| UMB0055 | Bladder (female) [no LUTS] | *L. jensenii* | GCF_007786035 | 100 | 3.6 |
| UMB0034 | Bladder (female) [no LUTS] | *L. jensenii* | GCF_007786085 | 100 | 3.6 |
| UMB1307 | Bladder (female) [UTI] | *L. jensenii* | GCF_007786135 | 100 | 3.6 |
| UMB1303 | Bladder (female) [UTI] | *L. jensenii* | GCF_007786145 | 84.8 | 8.1 |
| UMB1165 | Bladder (female) [UTI] | *L. jensenii* | GCF_007786155 | 100 | 3.6 |
| UMB8651 | Bladder (female) [OAB] | *L. jensenii* | GCF_008726325 | 100 | 3.6 |
| UMB4685 | Bladder (female) [OAB] | *L. jensenii* | GCF_008726555 | 100 | 3.6 |
| UMB3442 | Bladder (female) [OAB] | *L. jensenii* | GCF_008726585 | 100 | 3.6 |
| UMB0572 | Bladder (female) [UUI] | *L. jensenii* | GCF_008727025 | 100 | 3.6 |
| UMB0246 | Bladder (female) [OAB] | *L. jensenii* | GCF_008727085 | 100 | 3.6 |
| VA04-2An | Vagina [healthy] | *L. jensenii* | GCF_011029225 | 100 | 3.6 |
| UMB0847 | Bladder (female) [no LUTS] | *L. jensenii* | GCF_012029675 | 100 | 3.6 |
| UMB0836 | Bladder (female) [no LUTS] | *L. jensenii* | GCF_012029775 | 100 | 3.6 |
| JV-V16 | Vagina [healthy] | *L. mulieris* | GCF_000159335 | 100 | 0 |
| 27-2-CHN | Vagina [healthy] | *L. mulieris* | GCF_000161895 | 100 | 0 |
| 115-3-CHN | Vagina [healthy] | *L. mulieris* | GCF_000162435 | 100 | 0 |
| IM11 | Unknown | *L. mulieris* | GCF_001012655 | 100 | 0 |
| IM59 | Unknown | *L. mulieris* | GCF_001012675 | 100 | 0 |
| IM1 | Unknown | *L. mulieris* | GCF_001012735 | 100 | 0.1 |
| IM3 | Unknown | *L. mulieris* | GCF_001012745 | 100 | 0 |
| TL2937 | Feces [healthy] | *L. mulieris* | GCF_001742045 | 100 | 0.1 |
| c10Ua161M | Urinary tract (female) [healthy] | *L. mulieris* | GCF_007095465 | 100 | 3.6 |
| UMB1355 | Bladder (female) [UTI] | *L. mulieris* | GCF_007786095 | 100 | 0 |
| UMB8440 | Bladder (female) [OAB] | *L. mulieris* | GCF_008726405 | 100 | 3.6 |
| UMB4707 | Bladder (female) [OAB] | *L. mulieris* | GCF_008728065 | 100 | 0 |
| UMB0639 | Bladder (female) [OAB] | *L. mulieris* | GCF_008728115 | 100 | 0 |
| FDAARGOS_749 | Unknown | *L. mulieris* | GCF_009730255 | 100 | 0 |
| UMB7784 | Bladder (female) [rUTI] | *L. mulieris* | GCF_012102935 | 100 | 0 |
| UMB9245 | Bladder (female) [rUTI] | *L. mulieris* | GCF_012102955 | 100 | 0 |
| MGYG-HGUT-02313 | Human gut [unknown] | *L. mulieris* | GCF_902385715 | 100 | 0.1 |

For strains from our own collection (indicated by the strain designation “UMB”), the urinary tract symptom status is listed in brackets. Symptom status includes: OAB = overactive bladder symptoms; UTI = urinary tract infection; rUTI = recurrent UTI; UUI = urgency urinary incontinence; and no LUTS = no lower urinary tract symptoms.
