# Supplementary figures and images for: Comparative Genomic Study of Lactobacillus jensenii and the Newly Defined Lactobacillus mulieris Species Identifies Species-Specific Functionality
Source: mSphere. 2020 Aug 12;5(4):e00560-20. doi: 10.1128/mSphere.00560-20 (PMC7426171; doi:10.1128/mSphere.00560-20)

*L. jensenii*

*L. mulieris*

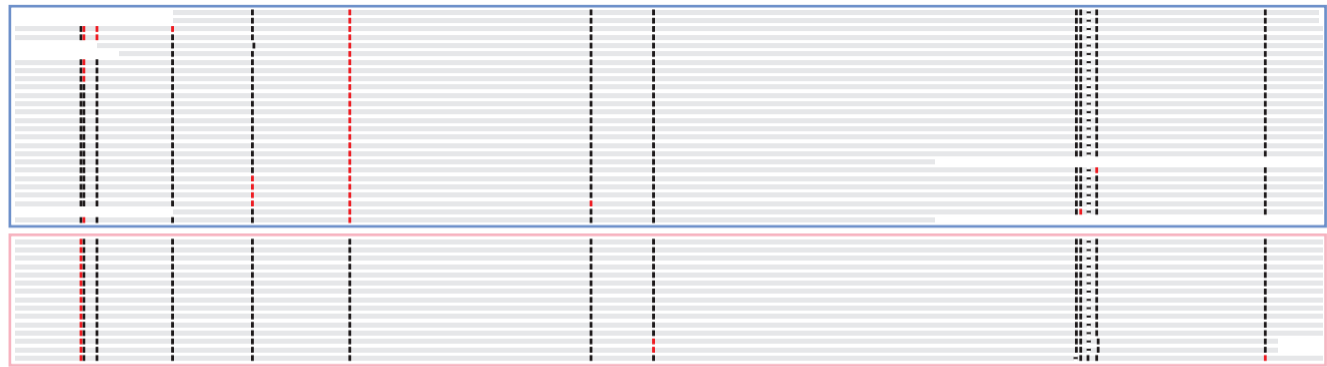

0

500

1000

1500

*Gene Sequence Position*

Lj=A; Lm=T  
Lg=A; Lc=C

Lj=A; Lm=C  
Lg=C; Lc=C

Supplement: FIG S1 [file mSphere.00560-20-sf001.pdf]
