# Supplementary material for: Comparative Genomic Study of Lactobacillus jensenii and the Newly Defined Lactobacillus mulieris Species Identifies Species-Specific Functionality
Source: mSphere. 2020 Aug 12;5(4):e00560-20. doi: 10.1128/mSphere.00560-20 (PMC7426171; doi:10.1128/mSphere.00560-20)
Supplement: TEXT S1 [file mSphere.00560-20-s0001.docx]

**Supplemental Methods**

**Genome Sequences**

In total, 43 *L. jensenii* and *L. mulieris* genomes were downloaded from NCBI. Species was determined according to (1). Metadata, including genome sequence accession numbers, can be found in **Table S1**. The site of isolation and host health status was determined from GenBank files followed by confirmation from the literature. For each genome, the CheckM Completeness and CheckM Contamination levels (2) were calculated using PATRIC (3), specifying the taxonomical group *L. jensenii* (Taxonomy ID: 109790).

**16S rRNA Gene Sequence Comparisons**

16S rRNA gene sequences were retrieved from the assembly or complete genome’s RefSeq annotation. To serve as an outgroup, the 16S sequences *L. gasseri* ATCC 33323 = JCM 1131 (NC_008530) and *L. crispatus* ST1 (NC_014106) were also retrieved from NCBI. The sequences were aligned using MAFFT v7.388 (4) through Geneious Prime 2019.1.1. The phylogenetic tree was derived using FastTree v2.1.11 (5) through Geneious. The resulting Newick format file was visualized using iTOL v5.5.1 (6).

**Pan and Core Genome Identification and Phylogeny**

The 43 genomes were analyzed using anvi’o version 5.1 (7). The core and accessory genes for both species were determined using the anvi-pan-genome function with the default minbit threshold of 0.5 and an mcl inflation value of 10. The clusters of homologous genes identified by anvi’o were examined further; the pairwise similarities for sequences within each cluster were calculated. On average, sequences within a cluster had a pairwise sequence identity of 86.8%. Python v3 was used to perform this analysis.

The set of single copy genes in the core genome were identified and the protein sequences were concatenated for each strain. The concatenated sequences were aligned using MUSCLE (8) and their phylogenetic tree was derived using IQTREE version 1.6.12 (9). The best fit substitution model was “JTTDCMut+F+R” according to BIC and selected within IQTREE using ModelFinder (10). This phylogeny was visualized using iTOL v5.5.1 (6). The pangenome visualization was generated using anvi’o version 6.2 (using the pangenome database created by the earlier version).

The core and accessory genes for each species were also determined using the anvi-pan-genome function with the default minbit threshold of 0.5 and an mcl inflation value of 10. Genes conserved amongst one species and absent from the genomes of the other species also were identified using anvi’o. These amino acid sequences were written to file and queried against the nr database via blastp to determine their protein product.

The pangenome curve was determined by parsing the anvi’o gene cluster data using Python v3.

**References:**

1. Rocha J, Botelho J, Ksiezarek M, Perovic SU, Machado M, Carriço JA, Pimentel LL, Salsinha S, Rodríguez-Alcalá LM, Pintado M, Ribeiro TG, Peixe L. 2020. Lactobacillus mulieris sp. nov., a new species of Lactobacillus delbrueckii group. Int J Syst Evol Microbiol 70:1522–1527.

2. Parks DH, Imelfort M, Skennerton CT, Hugenholtz P, Tyson GW. 2015. CheckM: assessing the quality of microbial genomes recovered from isolates, single cells, and metagenomes. Genome Res 25:1043–1055.

3. Wattam AR, Davis JJ, Assaf R, Boisvert S, Brettin T, Bun C, Conrad N, Dietrich EM, Disz T, Gabbard JL, Gerdes S, Henry CS, Kenyon RW, Machi D, Mao C, Nordberg EK, Olsen GJ, Murphy-Olson DE, Olson R, Overbeek R, Parrello B, Pusch GD, Shukla M, Vonstein V, Warren A, Xia F, Yoo H, Stevens RL. 2017. Improvements to PATRIC, the all-bacterial Bioinformatics Database and Analysis Resource Center. Nucleic Acids Res 45:D535–D542.

4. Katoh K, Standley DM. 2013. MAFFT multiple sequence alignment software version 7: improvements in performance and usability. Mol Biol Evol 30:772–780.

5. Price MN, Dehal PS, Arkin AP. 2010. FastTree 2 – Approximately Maximum-Likelihood Trees for Large Alignments. PLoS ONE 5:e9490.

6. Letunic I, Bork P. 2019. Interactive Tree Of Life (iTOL) v4: recent updates and new developments. Nucleic Acids Research 47:W256–W259.

7. Eren AM, Esen ÖC, Quince C, Vineis JH, Morrison HG, Sogin ML, Delmont TO. 2015. Anvi’o: an advanced analysis and visualization platform for ’omics data. PeerJ 3:e1319.

8. Edgar RC. 2004. MUSCLE: multiple sequence alignment with high accuracy and high throughput. Nucleic Acids Res 32:1792–1797.

9. Nguyen L-T, Schmidt HA, von Haeseler A, Minh BQ. 2015. IQ-TREE: a fast and effective stochastic algorithm for estimating maximum-likelihood phylogenies. Mol Biol Evol 32:268–274.

10. Kalyaanamoorthy S, Minh BQ, Wong TKF, von Haeseler A, Jermiin LS. 2017. ModelFinder: fast model selection for accurate phylogenetic estimates. Nat Methods 14:587–589.
