# Supplementary material for: Comparative Genomic Study of Lactobacillus jensenii and the Newly Defined Lactobacillus mulieris Species Identifies Species-Specific Functionality
Source: mSphere. 2020 Aug 12;5(4):e00560-20. doi: 10.1128/mSphere.00560-20 (PMC7426171; doi:10.1128/mSphere.00560-20)
Supplement: TABLE S2 [file mSphere.00560-20-st002.docx]

|  | c10Ua161M | **UMB7784** | JV-V16 | 27-2-CHN | 115-3-CHN | IM59 | IM1 | IM3 | TL2937 | UMB1355 | UMB8440 | UMB4707 | UMB639 | FDAARGOS_749 | MGYG-HGUT-02313 | IM11 | UMB9245 |
| --- | --- | --- | --- | --- | --- | --- | --- | --- | --- | --- | --- | --- | --- | --- | --- | --- | --- |
| c10Ua161M | * | 96.29 | 99.75 | 99.47 | 99.48 | 99.52 | 99.47 | 99.36 | 99.38 | 99.48 | 99.93 | 99.58 | 99.48 | 99.75 | 99.27 | 99.44 | 99.45 |
| **UMB7784** | 96.16 | * | 96.12 | 96.52 | 96.54 | 96.49 | 96.29 | 96.64 | 96.37 | 96.5 | 96.21 | 96.42 | 96.52 | 96.14 | 96.59 | 96.56 | 96.6 |
| JV-V16 | 99.91 | 96.32 | * | 99.66 | 99.66 | 99.71 | 99.58 | 99.53 | 99.55 | 99.66 | 99.93 | 99.7 | 99.65 | 99.99 | 99.29 | 99.42 | 99.5 |
| 27-2-CHN | 99.57 | 96.64 | 99.58 | * | 100 | 99.83 | 99.68 | 99.75 | 99.82 | 99.93 | 99.59 | 99.78 | 99.9 | 99.58 | 99.86 | 99.88 | 99.87 |
| 115-3-CHN | 99.46 | 96.64 | 99.47 | 99.99 | * | 99.82 | 99.61 | 99.8 | 99.76 | 99.91 | 99.47 | 99.72 | 99.88 | 99.48 | 99.86 | 99.85 | 99.87 |
| IM59 | 99.59 | 96.58 | 99.52 | 99.78 | 99.78 | * | 99.6 | 99.63 | 99.72 | 99.59 | 99.59 | 99.6 | 99.59 | 99.52 | 99.75 | 99.82 | 99.6 |
| IM1 | 99.63 | 96.57 | 99.61 | 99.74 | 99.72 | 99.76 | * | 99.62 | 99.59 | 99.75 | 99.61 | 99.75 | 99.73 | 99.61 | 99.37 | 99.56 | 99.63 |
| IM3 | 99.34 | 96.8 | 99.37 | 99.76 | 99.73 | 99.65 | 99.5 | * | 99.71 | 99.79 | 99.35 | 99.59 | 99.76 | 99.37 | 99.76 | 99.74 | 99.7 |
| TL2937 | 99.27 | 96.59 | 99.29 | 99.86 | 99.86 | 99.75 | 99.37 | 99.76 | * | 99.84 | 99.31 | 99.72 | 99.83 | 99.3 | 100 | 99.78 | 99.6 |
| UMB1355 | 99.49 | 96.72 | 99.44 | 99.86 | 99.85 | 99.68 | 99.58 | 99.6 | 99.69 | * | 99.49 | 99.58 | 99.83 | 99.44 | 99.84 | 99.87 | 99.73 |
| UMB8440 | 99.94 | 96.28 | 99.84 | 99.53 | 99.52 | 99.59 | 99.56 | 99.37 | 99.42 | 99.54 | * | 99.61 | 99.55 | 99.85 | 99.31 | 99.42 | 99.5 |
| UMB4707 | 99.62 | 96.55 | 99.59 | 99.72 | 99.73 | 99.76 | 99.64 | 99.55 | 99.73 | 99.63 | 99.62 | * | 99.62 | 99.59 | 99.72 | 99.78 | 99.47 |
| UMB639 | 99.55 | 96.69 | 99.53 | 99.89 | 99.87 | 99.75 | 99.64 | 99.71 | 99.86 | 99.89 | 99.57 | 99.58 | * | 99.54 | 99.86 | 99.89 | 99.75 |
| FDAARGOS_749 | 99.93 | 96.46 | 99.99 | 99.66 | 99.66 | 99.7 | 99.56 | 99.44 | 99.51 | 99.66 | 99.93 | 99.72 | 99.68 | * | 99.51 | 99.62 | 99.66 |
| MGYG-HGUT-02313 | 99.27 | 96.59 | 99.29 | 99.86 | 99.86 | 99.75 | 99.37 | 99.76 | 100 | 99.84 | 99.31 | 99.72 | 99.83 | 99.3 | * | 99.88 | 99.66 |
| IM11 | 99.44 | 96.56 | 99.42 | 99.88 | 99.85 | 99.82 | 99.56 | 99.74 | 99.78 | 99.87 | 99.42 | 99.78 | 99.89 | 99.42 | 99.78 | * | 99.9 |
| UMB9245 | 99.45 | 96.6 | 99.5 | 99.87 | 99.87 | 99.6 | 99.63 | 99.7 | 99.6 | 99.73 | 99.5 | 99.47 | 99.72 | 99.51 | 99.6 | 99.87 | * |
