# Supplementary material for: Comparative Genomic Study of Lactobacillus jensenii and the Newly Defined Lactobacillus mulieris Species Identifies Species-Specific Functionality
Source: mSphere. 2020 Aug 12;5(4):e00560-20. doi: 10.1128/mSphere.00560-20 (PMC7426171; doi:10.1128/mSphere.00560-20)
Supplement: TABLE S3 [file mSphere.00560-20-st003.docx]

| **Protein Product** | **Start Position** | **Stop Position** | **Product Accession** |
| --- | --- | --- | --- |
| sugar O-acetyltransferase | 71545 | 72138 | WP_006588339.1 |
| glycoside hydrolase family 32 protein | 73788 | 75074 | WP_006588338.1 |
| carbohydrate ABC transporter permease | 75074 | 75919 | WP_006585441.1 |
| sugar ABC transporter permease | 75922 | 76797 | WP_006585440.1 |
| sugar ABC transporter substrate-binding protein | 76818 | 78095 | WP_075362413.1 |
| LacI family DNA-binding transcriptional regulator | 78173 | 79156 | WP_006588337.1 |
| hypothetical protein | 134987 | 135799 | WP_006585391.1 |
| trehalose operon repressor | 295165 | 295878 | WP_006588288.1 |
| PTS glucose transporter subunit IIA | 296054 | 297964 | WP_006585286.1 |
| aminopeptidase C | 306762 | 308072 | WP_006588222.1 |
| hypothetical protein | 349458 | 349703 | WP_006585245.1 |
| cytochrome d ubiquinol oxidase subunit I | 509329 | 510762 | WP_006585099.1 |
| cytochrome d ubiquinol oxidase subunit II | 510763 | 511779 | WP_006585098.1 |
| thiol reductant ABC exporter subunit CydD | 511781 | 513496 | WP_006585097.1 |
| thiol reductant ABC exporter subunit CydC | 513489 | 515225 | WP_006585096.1 |
| polyprenyl synthetase family protein | 515230 | 516207 | WP_006588094.1 |
| 1,4-dihydroxy-2-naphthoate octaprenyltransferase | 516281 | 517180 | WP_006585094.1 |
| NAD(P)/FAD-dependent oxidoreductase | 517199 | 518410 | WP_006585093.1 |
| hypothetical protein | 681335 | 682624 | WP_006587936.1 |
| AMP-binding protein | 682630 | 685494 | WP_006587935.1 |
| PAS domain-containing protein | 762382 | 762825 | WP_006584898.1 |
| helix-turn-helix domain-containing protein | 826613 | 827650 | WP_006587885.1 |
| MFS transporter | 827754 | 829040 | WP_006587884.1 |
| family 78 glycoside hydrolase catalytic domain containing protein | 829043 | 830620 | WP_006587883.1 |
| MFS transporter | 830644 | 831876 | WP_006584853.1 |
| hypothetical protein | 928701 | 929156 | WP_006584775.1 |
| hypothetical protein | 929343 | 929576 | WP_006584774.1 |
| 1-deoxy-D-xylulose-5-phosphate synthase | 1052796 | 1054538 | WP_006584659.1 |
| GA module-containing protein | 1261750 | 1263900 | WP_075362495.1 |
| ribose transporter RbsU | 1300917 | 1301804 | WP_006584450.1 |
| restriction endonuclease subunit S | 1441809 | 1442927 | WP_006585687.1 |
| FAD-binding oxidoreductase | 1605747 | 1607153 | WP_006587763.1 |
