# Supplementary material for: Comparative Genomic Study of Lactobacillus jensenii and the Newly Defined Lactobacillus mulieris Species Identifies Species-Specific Functionality
Source: mSphere. 2020 Aug 12;5(4):e00560-20. doi: 10.1128/mSphere.00560-20 (PMC7426171; doi:10.1128/mSphere.00560-20)
Supplement: TABLE S4 [file mSphere.00560-20-st004.docx]

| **Protein Product** | **Contig** | **Start Position** | **Stop Position** | **Product Accession** |
| --- | --- | --- | --- | --- |
| YxeA family protein | 1 | 9719 | 10051 | WP_006586887.1 |
| ABC transporter ATP-binding protein | 1 | 10048 | 10716 | WP_006586888.1 |
| bacteriocin-associated protein | 1 | 10731 | 12710 | WP_006586889.1 |
| ATP-binding cassette domain-containing protein | 1 | 17179 | 18765 | WP_006586895.1 |
| ABC transporter ATP-binding protein | 1 | 18762 | 20339 | WP_034535762.1 |
| helix-turn-helix domain-containing protein | 1 | 20353 | 21171 | WP_143458368.1 |
| amino acid permease | 1 | 114428 | 115852 | WP_006586995.1 |
| MFS transporter | 1 | 117134 | 118327 | WP_006586997.1 |
| LPXTG cell wall anchor domain-containing protein | 1 | 160643 | 164437 | WP_162501143.1 |
| ammonium transporter | 2 | 24316 | 25530 | WP_006586098.1 |
| CPBP family intramembrane metalloprotease | 2 | 25784 | 26983 | WP_143458357.1 |
| helix-turn-helix transcriptional regulator | 2 | 38060 | 38908 | WP_006586086.1 |
| hypothetical protein | 2 | 39107 | 41197 | WP_006586084.1 |
| ATP-binding cassette domain-containing protein | 2 | 41190 | 41849 | WP_006586083.1 |
| helix-turn-helix domain-containing protein | 2 | 41834 | 42706 | WP_006586082.1 |
| MFS transporter | 2 | 42810 | 44066 | WP_006586081.1 |
| LysR family transcriptional regulator | 2 | 52319 | 53059 | WP_006586073.1 |
| tautomerase family protein | 2 | 53162 | 53560 | WP_006586072.1 |
| hypothetical protein | 2 | 53577 | 54209 | WP_006587531.1 |
| ArgE/DapE family deacylase | 2 | 62907 | 64052 | WP_006587528.1 |
| DUF2316 family protein | 2 | 123847 | 125355 | WP_006586010.1 |
| hypothetical protein | 2 | 132848 | 133087 | WP_006586002.1 |
| hypothetical protein | 3 | 17810 | 47137 | WP_006586641.1 |
| zinc metallopeptidase | 4 | 4708 | 5412 | WP_006586164.1 |
| LPXTG cell wall anchor domain-containing protein | 4 | 100654 | 101880 | WP_143458386.1 |
| hypothetical protein | 4 | 117704 | 118024 | WP_006586272.1 |
| amidohydrolase family protein | 4 | 119095 | 120006 | WP_006586274.1 |
| MerR family DNA-binding transcriptional regulator | 4 | 120062 | 120469 | WP_006586275.1 |
| aldo/keto reductase | 4 | 120485 | 121423 | WP_006586276.1 |
| DUF2255 family protein | 4 | 121445 | 121828 | WP_006586277.1 |
| hypothetical protein | 4 | 137914 | 141039 | WP_006586294.1 |
| hypothetical protein | 5 | 82355 | 82501 | WP_022091177.1 |
| LacI family DNA-binding transcriptional regulator | 6 | 50099 | 51121 | WP_143458486.1 |
| hypothetical protein | 6 | 111667 | 112500 | WP_006586350.1 |
| hypothetical protein | 7 | 28065 | 29942 | WP_143458450.1 |
| glycoside hydrolase family 68 protein | 8 | 51412 | 53760 | WP_006587640.1 |
| ribokinase | 9 | 56072 | 56995 | WP_006587086.1 |
| GNAT family N-acetyltransferase | 9 | 88480 | 89001 | WP_006587116.1 |
| LPXTG cell wall anchor domain-containing protein | 9 | 89096 | 92065 | WP_143460224.1 |
| 30S ribosomal protein S14 rpsN | 10 | 5140 | 5409 | WP_006586691.1 |
| hypothetical protein | 10 | 5424 | 5849 | WP_006586692.1 |
| putative metal homeostasis protein | 10 | 7599 | 7715 | WP_022091160.1 |
| hypothetical protein | 10 | 33882 | 34862 | WP_006586721.1 |
| FAD-dependent oxidoreductase | 10 | 65725 | 67095 | WP_006586751.1 |
| hypothetical protein | 10 | 67328 | 68389 | WP_006586752.1 |
| L-2-hydroxyisocaproate dehydrogenase | 12 | 2577 | 3449 | WP_022091329.1 |
| GntR family transcriptional regulator | 12 | 5018 | 5719 | WP_006586313.1 |
| DUF3737 family protein | 12 | 34506 | 35378 | WP_006586336.1 |
| pyridoxal phosphate-dependent aminotransferase | 12 | 35378 | 36541 | WP_006586337.1 |
| M42 family metallopeptidase | 13 | 5898 | 6980 | WP_006587121.1 |
| FIVAR domain-containing protein | 13 | 14942 | 19969 | WP_143458494.1 |
| amidohydrolase family protein | 13 | 28327 | 29682 | WP_006587139.1 |
| purine/pyrimidine permease | 13 | 29693 | 31015 | WP_006587140.1 |
| hypothetical protein | 16 | 3795 | 4223 | WP_006585826.1 |
| TetR/AcrR family transcriptional regulator | 17 | 14461 | 15018 | WP_006585948.1 |
| glycerophosphodiester phosphodiesterase | 17 | 21358 | 22302 | WP_143458502.1 |
| hypothetical protein | 18 | 2075 | 2437 | WP_006587562.1 |
| nucleoside deaminase | 18 | 18639 | 19091 | WP_006586135.1 |
| GNAT family N-acetyltransferase | 19 | 4902 | 5408 | WP_006585787.1 |
| YSIRK-type signal peptide-containing protein | 19 | 11036 | 17829 | WP_143460227.1 |
| amino acid permease | 23 | 215 | 1675 | WP_167494287.1 |
| ferrochelatase | 27 | 1529 | 2467 | WP_006587679.1 |
